# Platelet-rich plasma combined with injectable hyaluronic acid hydrogel for porcine cartilage regeneration: a 6-month follow-up

**DOI:** 10.1093/rb/rbz039

**Published:** 2019-11-21

**Authors:** Wenqiang Yan, Xingquan Xu, Qian Xu, Ziying Sun, Qing Jiang, Dongquan Shi

**Affiliations:** 1 State Key Laboratory of Pharmaceutical Biotechnology, Department of Sports Medicine and Adult Reconstructive Surgery, Nanjing Drum Tower Hospital, The Affiliated Hospital of Nanjing University Medical School, 321 Zhongshan Road, Nanjing 210008, Jiangsu, China; 2 Laboratory for Bone and Joint Disease, Model Animal Research Center (MARC), Nanjing University, Nanjing 210093, Jiangsu, China

**Keywords:** platelet-rich plasma, hyaluronic acid hydrogel, cartilage repair, regenerative medicine

## Abstract

Based on our previous study, the utilization of an ultraviolet light photo-cross-linkable hyaluronic acid (HA) hydrogel integrated with a small molecule kartogenin-encapsulated nanoparticles obtained good reconstruction of osteochondral defects in a rabbit model, indicating the superiority of injectable hydrogel-based scaffolds in cartilage tissue engineering. Platelet-rich plasma (PRP), rich in various growth factors, proteins and cytokines, is considered to facilitate cartilage healing by stimulating cell proliferation and inducing chondrogenesis in cartilage defect site. The aim of this study was to test the therapeutic feasibility of autologous PRP combined with injectable HA hydrogel on cartilage repair. The focal cartilage defects with different critical sizes in the medial femoral condyle of a porcine model were used. At 6 months, the minipigs were sacrificed for assessment of macroscopic appearance, magnetic resonance imaging, micro-computed tomography, histology staining and biomechanics. The HA hydrogel combined with PRP-treated group showed more hyaline-like cartilage exhibited by macroscopic appearance and histological staining in terms of extracellular matrix and type II collagen without formation of hypertrophic cartilage, indicating its capacity to improve cartilage healing in the minipig model evaluated at 6 months, with full-thickness cartilage defect of 8.5 mm diameter and osteochondral defect of 6.5 mm diameter, 5 mm depth exhibiting apparent regeneration.

## Introduction

Articular cartilage defects have long-term deleterious influence [1] due to its limited spontaneous healing potential [2]. The existing strategies for articular cartilage regeneration involving nonsurgical treatment, microfracture, intra-articular injection, scaffold implantation with or without cell seeding have not yet obtained normal hyaline cartilage with appropriate mechanical properties and zonal organization of intact cartilage [3–5]. Moreover, the following characteristics including cost-efficient and easy accessible of cartilage repair techniques were required for its widespread application in clinic. The facilitated endogenous repair of cartilage was to stimulate the reparative potential of endogenous tissue, which can be realized by the addition of exogenous substances, such as autologous PRP and hydrated hydrogel implantation [6]. 

Platelet-rich plasma (PRP), rich in various growth factors, proteins and cytokines, is considered to initiate and regulate cartilage healing by stimulating cell proliferation and inducing chondrogenesis in cartilage defect site [7]. In addition, the potential effectiveness and good biocompatibility of PRP on cartilage repair have been demonstrated by many studies [8, 9]. Hyaluronic acid (HA) hydrogels can be a good choice owing to its highly hydrated, good biocompatibility, chemical–physical properties, capability to be degraded into safe products, and ability to simulate the extracellular matrix (ECM) environment [10, 11]. Our previous study has demonstrated that the utilization of an ultraviolet (UV) light photo-cross-linkable HA hydrogel integrated with a small molecule kartogenin-encapsulated nanoparticles obtained good reconstruction of osteochondral defects in a rabbit model [12].

To further test the therapeutic feasibility of PRP combined with HA hydrogel on cartilage repair, the focal cartilage defects with critical size in the medial femoral condyle of a porcine model were used. Moreover, the defects with different sizes were created to assess which defect size it is suitable for repairing in future clinical applications. We hypothesized that the HA hydrogel was responsible for recruiting endogenous cells, including mesenchymal stem cells and cartilage progenitor cells and PRP provided necessary growth factors for inducing chondrogenesis.

## Materials and methods

### Preparation of autologous PRP

**Figure 1:**
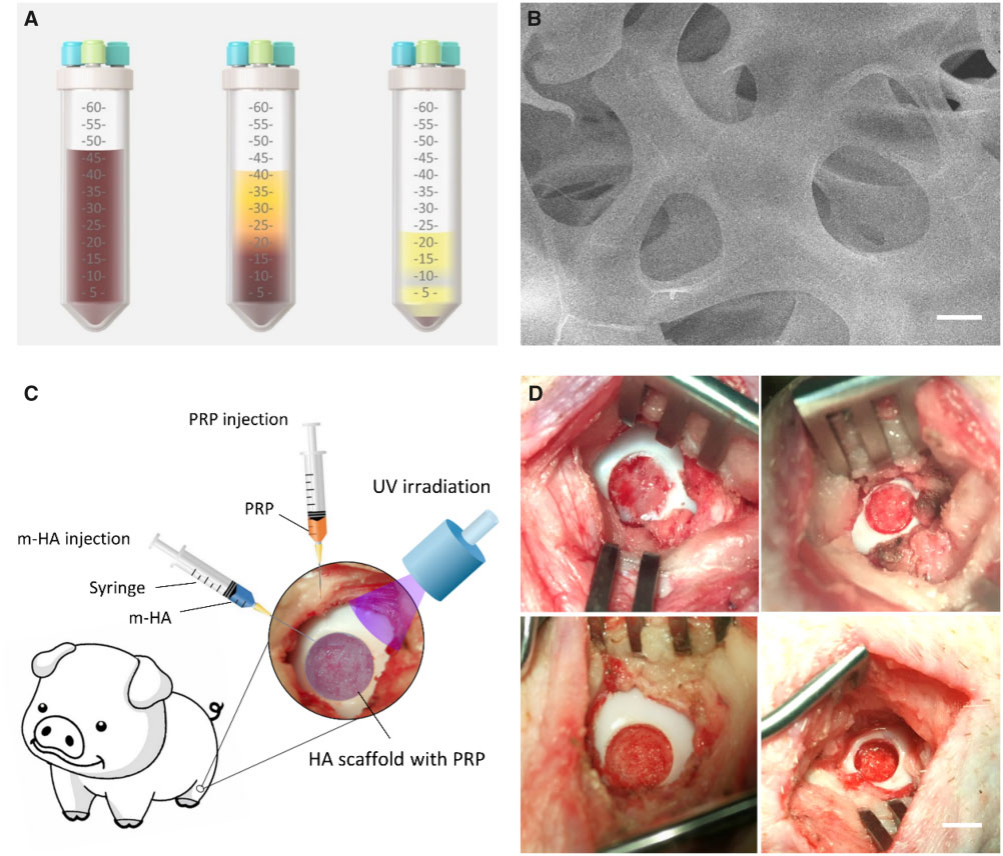
(**A**) Schematic of the preparation of autologous PRP (left, collection of whole blood; middle, first centrifugation; right, second centrifugation). (**B**) The SEM of m-HA hydrogel. Scale bar: 5 µm. (**C**) Schematic of surgical procedure. (**D**) The demonstration of FT cartilage defect and osteochondral defect in the medial femoral condyle. Scale bar: 5 mm (top, FT cartilage defect; bottom, osteochondral defect)

All animal procedures were approved by Institutional Animal Care and Use Committee of Nanjing Drum Tower Hospital, Medical School, Nanjing University. The minipigs were treated with xylazine hydrochloride (0.2 ml/kg), droperidol (0.2 mg/kg) and atropine (0.02 ml/kg) by intramuscular injection. Intravenous infusion of propofol was used to maintain anesthesia. Then the minipigs were put in a supine position after general anesthetization and 45 ml whole blood was collected from the precaval vein and mixed with 5 ml sodium citrate solution to prevent blood clotting. Then a commercial PRP extraction suit (WEGO Biotech Co, Weihai, China) was used to extract PRP concentrate. The tube was centrifuged at room temperature for 10 min at 2400 rpm. The blood was divided into three layers: plasma in the first layer, a buffy coat in the middle layer and red blood cells in the third layer. The red blood cells were removed through the pipe within the tube. Then second centrifugation was performed in the same tube at room temperature for 10 min at 2400 rpm (Fig. 1A). The supernatant plasma was removed through the intrinsic pipe and 1 ml of concentrated PRP was pipetted and injected into a sterile tube for further intra-articular injection. The concentrated PRP and whole blood were detected by hematology analyzer for white blood cell count and platelet count. For our study, the mean platelet count (5549.0 × 10^9^/l) of PRP was obviously (15-fold) greater than that of whole blood (373.0 × 10^9^/l). The mean white blood cell count (33.5 × 10^9^/l) of PRP was also greater than that of whole blood (19.5 × 10^9^/l).

### Preparation and characterization of photo-polymerizable HA hydrogel

The preparation of modified HA was performed by the reaction with methacrylic anhydride (MA) to obtain the double bond. Two grams of HA (Sigma-Aldrich) was dissolved in distilled (DI) water of 100 ml by stirring in a low temperature overnight, then 1.6 ml of MA was added into the prepared HA solution. The 5 N NaOH solution was used to maintain the pH of reaction system between 8 and 9, followed by continuous stirring for 24 h at 4°C. Then, the m-HA solution was obtained after precipitation in acetone, wash with ethanol and dissolution in DI water. The purification of m-HA was realized by performing dialysis against DI water for 48 h, followed by the acquisition of purified m-HA with a yield of 87.5% after lyophilization and characterization by 1H NMR (Varian Gemini 2300). The degree of modification (DM) was determined to be approximate 15% by performing the comparison between the ratio of the areas under proton peaks at 5.74 and 6.17 ppm (methacrylate protons) and the peak at 1.99 ppm (*N*-acetyl glucosamine of HA) after a standard deconvolution algorithm for separating closely spaced peaks was performed: m-HA:1H NMR (D_2_O, 300 MHz, δ ppm):1.85–1.96(m,3H,CH_2_=C(CH_3_)CO), 1.99(s,3H,NHCOCH_3_), 5.74(s,1H,CH_1_H_2_=C(CH_3_)CO), 6.17(s,1H,CH_1_H_2_=C(CH_3_)CO).

The photopolymerization was performed to form hydrogel via m-HA (2%, w/v), *N*,*N*-methylenebis(acrylamide) (MBA) (MBA/m-HA, 0.5:1, w:w) and photoinitiator (Irgacure 2959; 0.1%, w/v) followed by UV radiation for 1 min using a BlueWave 75 UV Curing Spot Lamp (DYMAX). Prior to the SEM scanning, the hydrogels were lyophilized first, then the microstructure of the hydrogel was acquired using a Quanta Scanning Electron Microscope (Quata 200, FEI) at 20 kV (Fig. 1B).

### Study design and surgical procedure

Twenty-four skeletally mature minipigs (Bama minipig, female, 40 ± 10 kg) were randomly divided into three groups, m-HA + PRP-treated group, m-HA-treated group and blank group. m-HA was injected into the defect site, then the hydrogel was formed via UV irradiation for 1 min followed by PRP injection into the joint cavity in the m-HA + PRP-treated group. Pure m-HA was injected into the defect site and irradiated to form the hydrogel in the m-HA-treated group. The defects of the blank group were left untreated (Fig. 1C). All minipigs were treated with xylazine hydrochloride (0.2 ml/kg), droperidol (0.2 mg/kg) and atropine (0.02 ml/kg) by intramuscular injection. Intravenous infusion of propofol was used to maintain anesthesia. Then, the minipigs were put in a supine position after general anesthetization. The endotracheal intubation and mechanical ventilation with a tidal volume of 300 ml were prepared to maintain normal ventilation. A medial para-patellar incision was used to perform the dislocation of patellar and exposure of the femoral condyle articular surface under sterile conditions. Four kinds of defect size were created in the medial femoral condyle weight bearing area in both knees by using an osteochondral transplantation instrumentation. Full-thickness (FT) cartilage defects with the subchondral bone preserved (6.5 mm in diameter, 8.5 mm in diameter) and osteochondral defects with the subchondral bone removed (6.5 mm in diameter, 5 mm in depth; 8.5 mm in diameter, 5 mm in depth) were included in this experiment (Fig. 1D). The prevention of infection was performed by injecting penicillin sodium and the minipigs were allowed to move freely in a comfortable environment.

### Macroscopic evaluation 

After a 6-month follow-up, the animals were sacrificed by intravenous propofol overdose. The regenerated tissue of the knee joints were exposed and photographed.

The repaired tissue was evaluated referring to the International Cartilage Repair Society (ICRS) macroscopic score (Table 1) containing three categories: macroscopic appearance, degree of defect repair and integration to board zone. The scoring was finished by three different observers.

**Table 1:** ICRS macroscopic evaluation of cartilage repair

Cartilage repair assessment ICRS	Points
Degree of defect repair	
In level with surrounding cartilage	4
75% repair of defect depth	3
50% repair of defect depth	2
25% repair of defect depth	1
0% repair of defect depth	0
Integration to border zone	
Complete integration with surrounding cartilage	4
Demarcating border < 1 mm	3
Three-fourths of graft integrated with surrounding	2
With a notable border >1 mm width and half of graft integrated with surrounding	1
From no contact to one-fourth of graft integrated with surrounding cartilage	0
Macroscopic appearance	
Intact smooth surface	4
Fibrillated surface	3
Small, scattered fissures or cracks	2
Several, small or few but large fissures	1
Total degeneration of grafted area	0
Overall repair assessment	
Grade I: normal	2
Grade II: nearly normal	1-8
Grade III: abnormal	7-4
Grade IV: severely abnormal	3-1

### Magnetic resonance imaging acquisition and assessment

The magnetic resonance imaging (MRI) of knee joints was obtained with a 3.0-T MRI Scanner (United Imaging, China). The high-resolution MRI images were obtained in the sagittal plane. The images of the maximum cross-section of defect area were selected to assess the repaired cartilage. The repaired tissue was assessed referring to the International Cartilage Repair Society (ICRS) Whole-Organ MRI Score (WORMS) of the knee in OA. The scoring was finished by three different observers.

### Micro-computed tomography analysis

For assessment of the subchondral bone formation, the specimens harvested from the osteochondral defect groups were wrapped with parafilm, and scanned with a µCT scanner (uCT80, SCANCO Medical AG, Switzerland) with unfiltered X-ray beam, 90 kV of source voltage, 160 µA of tube current and 80 µm of voxel resolution. Cross-sectional images were reconstructed with SCANCO Medical software. Then, the images of the maximum cross-section of defect area were selected to assess the reconstruction of subchondral bone. The rectangular region of interest (ROI) covering the operated area was selected on the reconstructed images for further analysis. For quantification of the regenerated bone, the parameters including percentage of bone volume (BV/TV), bone mineral density (BMD) and trabecular thickness (Tb.Th) were measured and calculated.

### Histology evaluation

The samples were embedded in paraffin after fixation in 10% formalin for at least 24 h and decalcification in 15% EDTA for 60 days. Then, the sections with 3 µm of thickness were stained with HE staining, toluidine blue and Safranin O/fast green staining to assess the morphology and glycosaminoglycan content. The observation was performed using a light microscope (Olympus, Japan). The regenerated tissue was scored by three different investigators using the scoring system of ICRS score including the parameters of cartilage thickness, surface regularity, integration with adjacent host cartilage, cell morphology and matrix staining.

### Immunohistochemistry evaluation

For immunohistochemical evaluation, primary antibody, rabbit anti-col II (abcam, ab34712), rabbit anti-col I (abcam, ab34710) and mouse anti-col X (abcam, ab49945) were used in the present study. The goat anti-rabbit IgG H&L (HRP) (abcam, ab6721) was used as secondary antibody. The sections were first incubated with 0.4% pepsin (Roche) at 37°C for 1 h for antigen retrieval. The 3% H_2_O_2_ solution was used to block the endogenous peroxidase, and 1% BSA (Sigma) was applied to block nonspecific protein binding. The sections were first incubated with primary antibody overnight at 4°C, followed by the incubation with secondary antibody for 1 h at room temperature. Then, the color was developed by using the DAB substrate system.

### Biomechanical test

The nanoindentation test was performed to analyze the biomechanical properties of repaired tissues. Before testing, all samples were harvested from the regenerated area using a trepan and immersed into PBS solution to maintain hydration at 4°C. All the indentation tests were performed by Agilent Nano Indenter G200 (Agilent Inc., California, USA) in five regions of each sample. A trapezoidal load function was applied to each indent site with loading (10 s), hold (2 s), and unloading (10 s). The indentations were performed with a force-controlled pattern to a maximum indentation depth of 500 nm. The linear fit to load–displacement data was used to calculate the hardness and reduced modulus.

### Statistical analysis

Descriptive statistics was used to summarize the data. All data were described as means and standard deviations (SD). Statistical analysis was performed with IBM SPSS Statistics 16 (IBM Corporation, NY, USA). The one-way ANOVA analysis with Bonferroni’s multiple comparison test was used for the statistical analysis. A value of *P* < 0.05 was considered to be statistically significant difference for all tests.

## Results

### Macroscopic appearance and scoring

**Figure 2:**
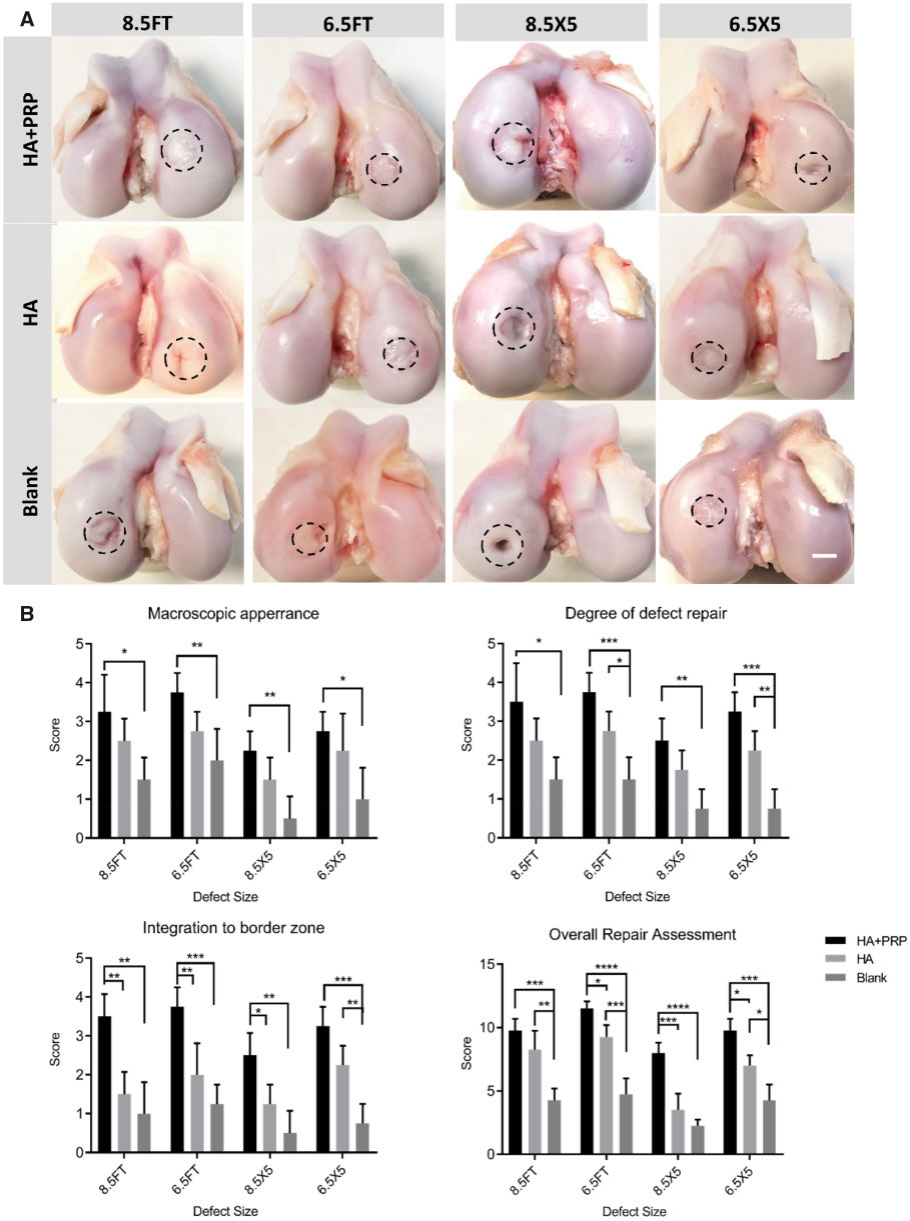
(**A**) Representative photos of macroscopic appearance of the repaired area. FT, full-thickness; HA, hyaluronic acid hydrogel; PRP, platelet-rich plasma; 8.5FT, 8.5 mm in diameter FT cartilage defect; 6.5FT, 6.5 mm in diameter FT cartilage defect; 8.5X5, 8.5 mm in diameter and 5 mm in depth osteochondral defect; 6.5X5, 6.5 mm in diameter and 5 mm in depth osteochondral defect; scale bar: 5 mm. The dashed circles indicated original injury boundaries. (**B**) ICRS macroscopic evaluation of cartilage repair. The results were represented as mean ± standard deviation. *P <* *0.05; **P <* *0.01; ***P <* *0.0005; ****P <* *0.0001

At 6 months after surgery, no signs of osteophyte formation, synovial hypertrophy and meniscal tears occurred in all minipigs. Either FT cartilage defect group or osteochondral defect group showed some extent of cartilage regeneration ranging from mild to distinct. For each defect size, the HA+PRP-treated group exhibited apparent regeneration with smooth articular cartilage surface and more transparent hyaline-like tissue integrated well with surrounding cartilage (Fig. 2A). In contrast, the HA treated or blank group exhibited inferior regeneration with recognizable defect margin, low level hyaline-like tissue or fibrous tissue and cracks. For FT cartilage defect with 8.5 mm in diameter, the HA+PRP-treated defects exhibited apparent cartilage regeneration. Moreover, for osteochondral defects with 6.5 × 5 mm, the HA+PRP-treated defects were in level with the adjacent cartilage (Fig. 2A). The results of ICRS macroscopic evaluation scoring were consistent with the gross appearance in terms of macroscopic appearance, degree of defect repair, integration to border zone and overall repair assessment (Fig. 2B).

### MRI assessment and scoring

**Figure 3:**
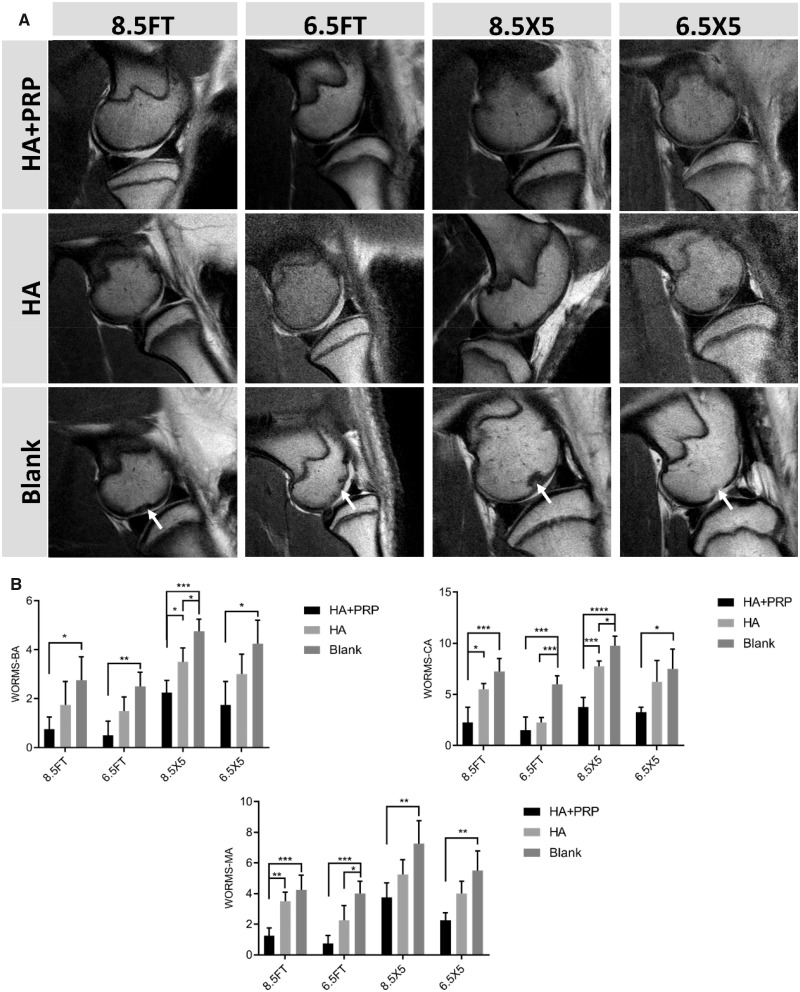
(**A**) The high-resolution magnetic resonance imaging of articular cartilage in the sagittal plane. From left to right, the arrows indicated abnormal subchondral bone marrow signal, diffused cartilage erosion, subchondral bone defect and cartilage defect. (**B**) The MRI ICRS-WORMS scoring of repaired tissue. WORMS-BA, WORMS-Bone attrition; WORMS-CA, WORMS-Cartilage; WORMS-MA, WORMS-Marrow abnormality. The results were represented as mean ± standard deviation. *P <* *0.05; **P <* *0.01; ***P <* *0.0005; ****P <* *0.0001

The high-resolution MRI was performed at 6 months after operation and the ICRS-WORMS scoring of the knee based on MRI images was used to evaluate the regeneration and OA progression. For FT cartilage defect group, the repaired area of HA+PRP-treated group showed similar signal compared to the adjacent native cartilage and no signs of subchondral bone marrow edema. In contrast, the HA treated or blank group exhibited signals of subchondral bone marrow edema or diffused cartilage erosion. For osteochondral defect group, the cartilage layer and subchondral bone layer of HA+PRP-treated group exhibited similar signal with the adjacent native tissue. However, the abnormal subchondral bone signal still occurred in the HA-treated group indicating inferior cartilage or subchondral bone repair. The significant subchondral bone attrition was observed in the Blank group (Fig. 3A). The results of ICRS-WORMS scoring were consistent with the MRI images in terms of bone attrition, cartilage and marrow abnormality (Fig. 3B).

### µCT analysis

**Figure 4:**
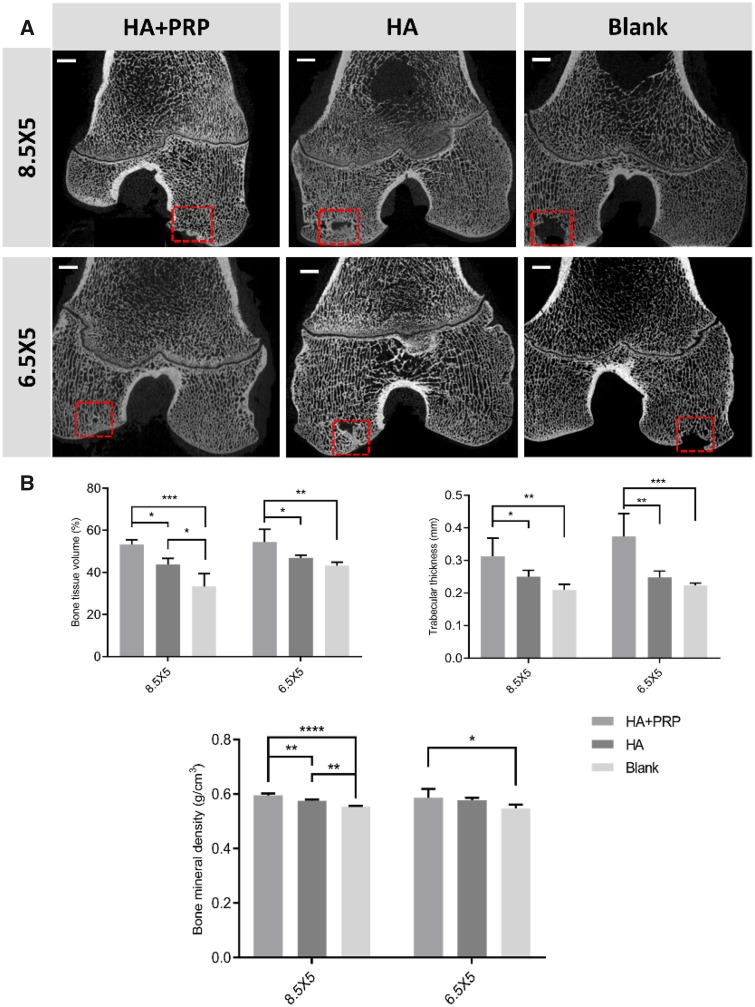
The evaluation of subchondral bone reconstruction of osteochondral defects by µCT images (**A**) and the quantitative analysis of bone tissue volume (%); Tb.Th (mm) and bone mineral density (g/cm^3^). (**B**) The results were represented as mean ± standard deviation. *P <* *0.05; **P <* *0.01; ***P <* *0.0005; ****P <* *0.0001. Scale bar: 3 mm

The micro-computed tomography (µCT) analysis was used to evaluate the subchondral bone reconstruction of osteochondral defects and quantitative analysis of bone tissue volume (%); Tb.Th (mm) and bone mineral density (g/cm^3^). All osteochondral defects had developed some extent of subchondral bone reconstruction ranging from partial to intact. The HA+PRP group exhibited intact subchondral bone reconstruction with continuous architecture and integration with the adjacent bone. The HA-treated group showed inferior subchondral bone reconstruction with cavity remained in the central hole. The worst subchondral bone reconstruction occurred in the Blank group (Fig. 4A). As was consistent with the micro-CT images, the HA+PRP group had significant higher bone tissue volume, Tb.Th and bone mineral density (Fig. 4B).

### Histological and immunohistochemical analysis

**Figure 5:**
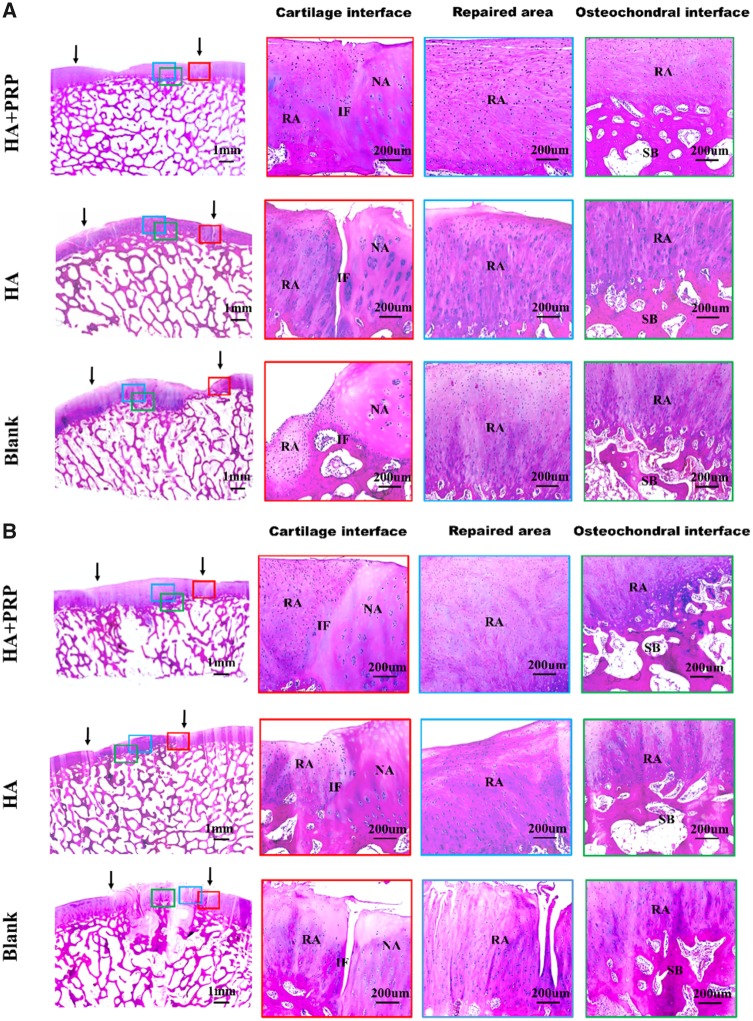
HE staining of repaired tissue showing the cartilage interface, repaired area and osteochondral interface. (**A**) FT cartilage defects with 8.5 mm in diameter. (**B**) FT cartilage defects with 6.5 mm in diameter. NA, native area; IF, interface; RA, repaired area; SB, subchondral bone. Black arrows indicated repaired area

**Figure 6:**
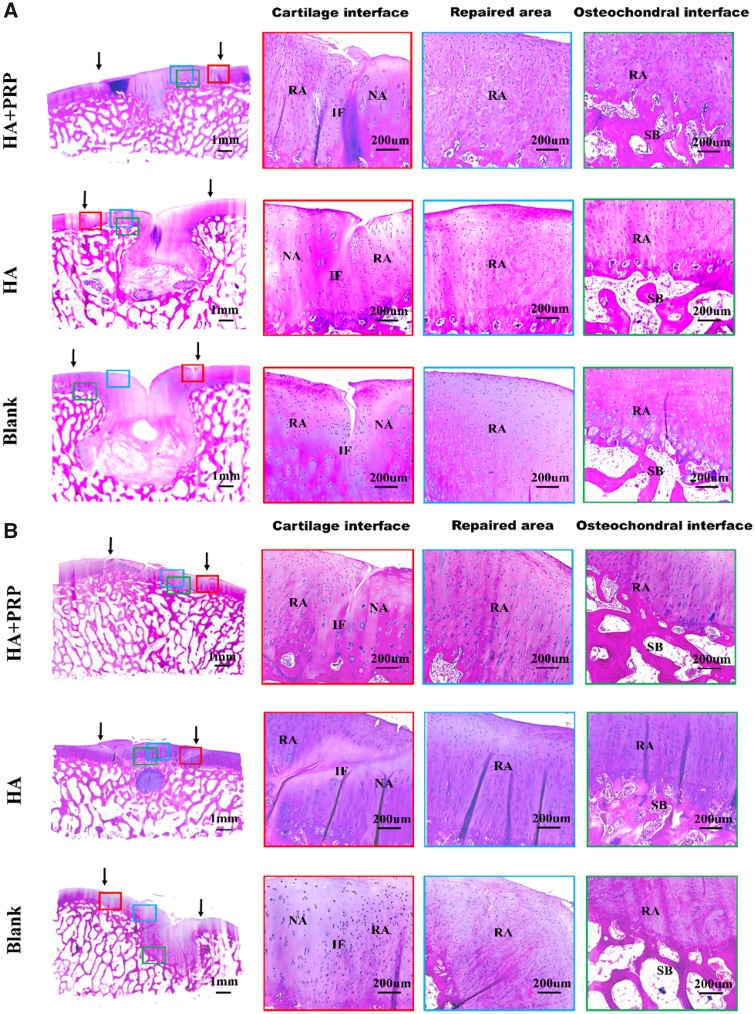
HE staining of repaired tissue showing the cartilage interface, repaired area and osteochondral interface. (**A**) Osteochondral defects with 8.5 mm in diameter and 5 mm in depth. (**B**) Osteochondral defects with 6.5 mm in diameter and 5 mm in depth. NA, native area; IF, interface; RA, repaired area; SB, subchondral bone. Black arrows indicated repaired area

The morphology and structure of repaired tissue were assessed by HE staining (Figs 5 and 6). No matter in FT cartilage defects or osteochondral defects, the HA+PRP-treated group exhibited the best regeneration compared to the control groups in terms of smooth surface, columnar cell distribution, cell viability and subchondral bone remodeling.

**Figure 7:**
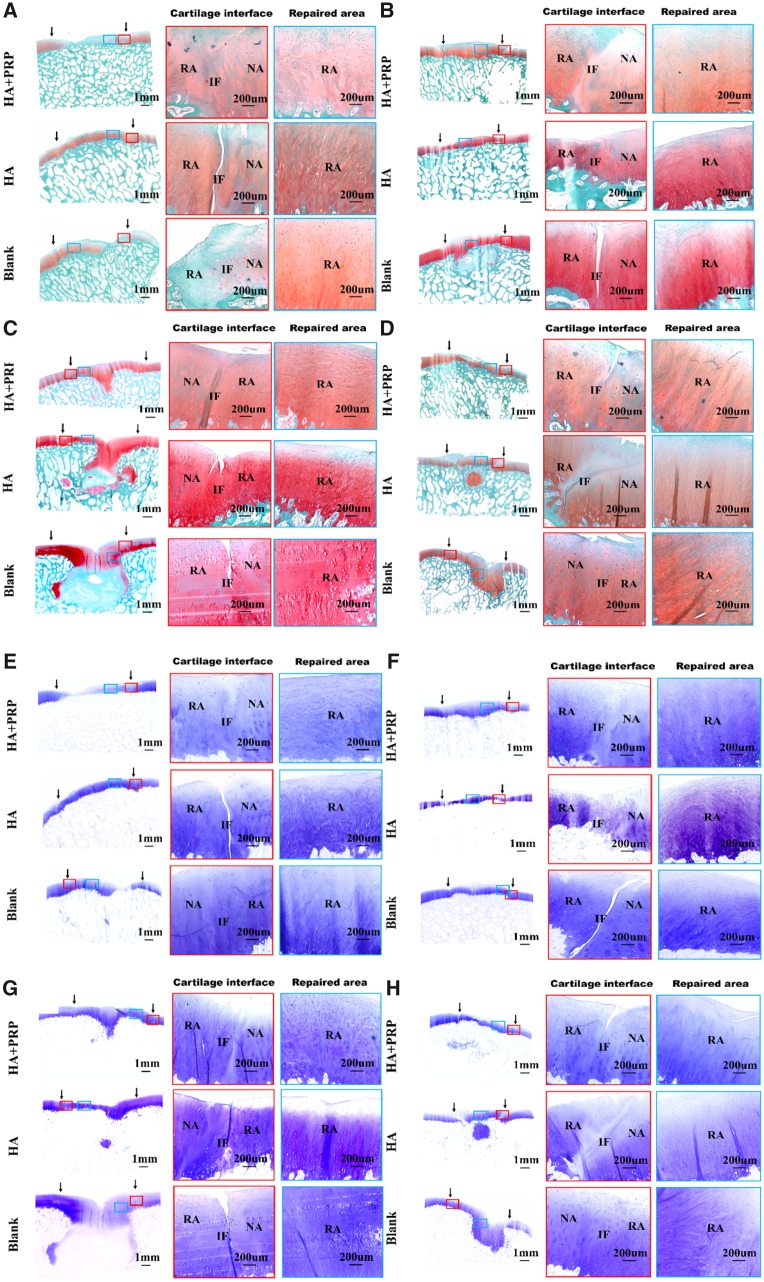
Safranin O/fast green staining and toluidine blue staining of repaired tissue showing the cartilage interface and repaired area. (**A–D**) Safranin O/fast green staining. (**E–H**) Toluidine blue staining. (A, E) FT cartilage defects with 8.5 mm in diameter. (B, F) FT cartilage defects with 6.5 mm in diameter. (C, G) Osteochondral defects with 8.5 mm in diameter and 5 mm in depth. (D, H) Osteochondral defects with 6.5 mm in diameter and 5 mm in depth. NA, native area; IF, interface; RA, repaired area. Black arrows indicated repaired area

**Figure 8:**
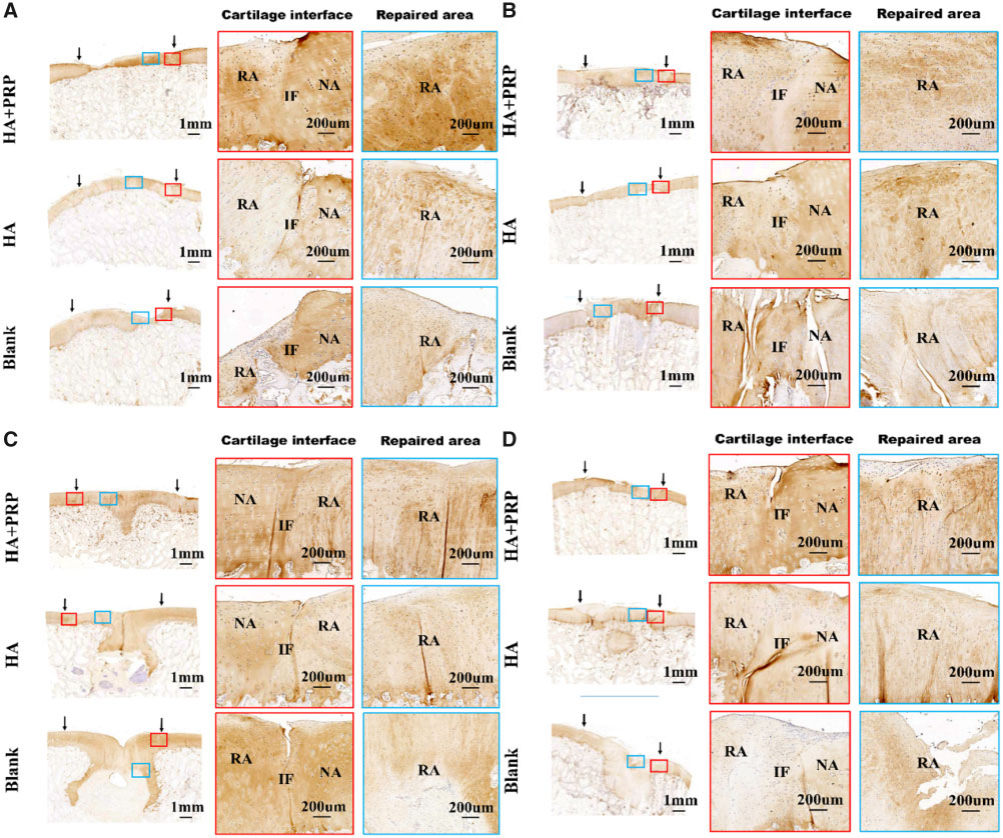
Immunohistochemical staining of repaired tissue showing the type II collagen. (**A**) FT cartilage defects with 8.5 mm in diameter. (**B**) FT cartilage defects with 6.5 mm in diameter. (**C**) Osteochondral defects with 8.5 mm in diameter and 5 mm in depth. (**D**) Osteochondral defects with 6.5 mm in diameter and 5 mm in depth. NA, native area; IF, interface; RA, repaired area. Black arrows indicated repaired area

**Figure 9:**
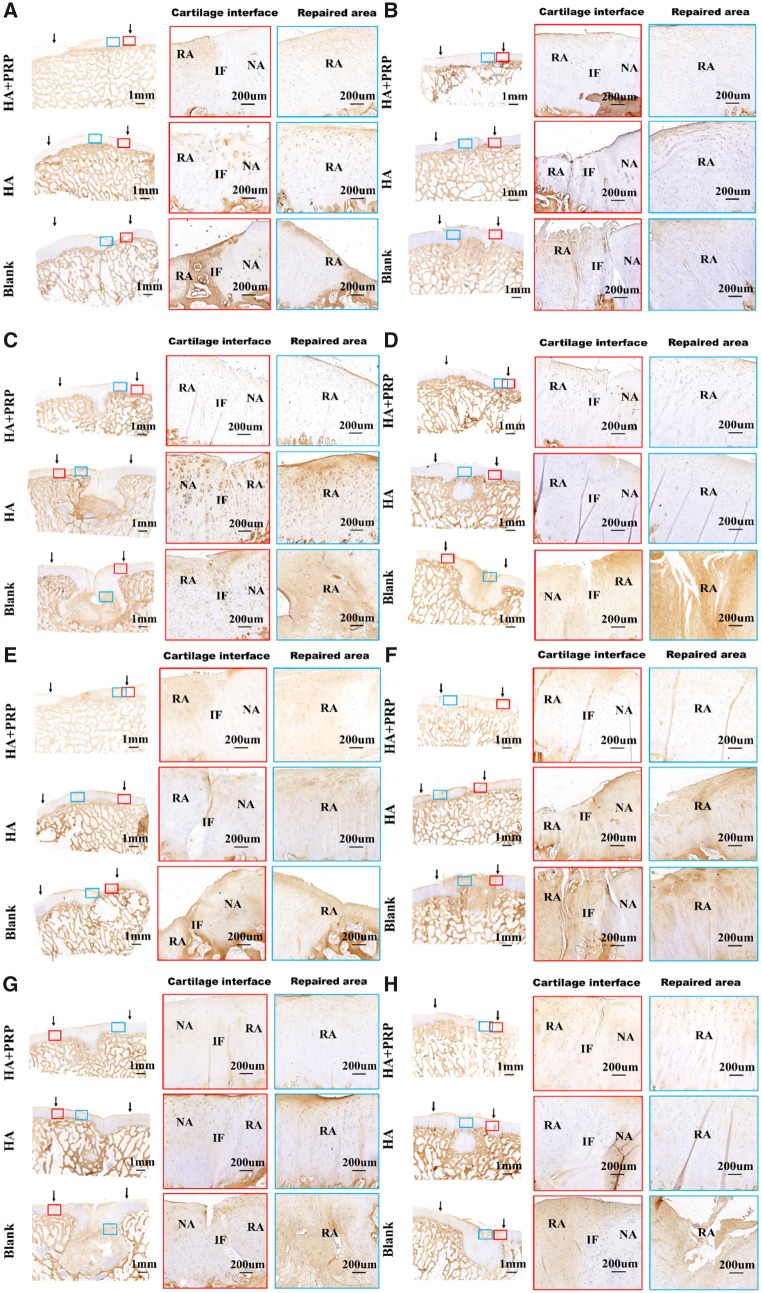
Immunohistochemical staining of repaired tissue showing the type I and type collagen. (**A–D**) Immunohistochemical staining of type I collagen; (**E–H**) immunohistochemical staining of type collagen. (A, E) FT cartilage defects with 8.5 mm in diameter. (B, F) FT cartilage defects with 6.5 mm in diameter. (C, G) Osteochondral defects with 8.5 mm in diameter and 5 mm in depth. (D, H) Osteochondral defects with 6.5 mm in diameter and 5 mm in depth. NA, native area; IF, interface; RA, repaired area. Black arrows indicated repaired area

**Figure 10:**
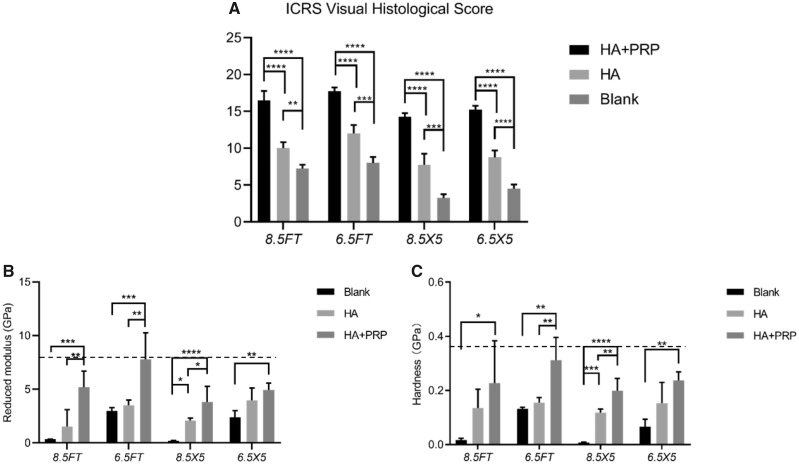
ICRS visual histological score of repaired tissue and nanoindentation test of repaired tissue. (**A**) ICRS visual histological score of repaired tissue. (**B**) Reduced modulus and (**C**) hardness of repaired cartilage. The dotted horizontal line represented the mechanical properties of native intact cartilage. The results were demonstrated by mean ± standard deviation. *P <* *0.05; **P <* *0.01; ***P <* *0.0005; ****P <* *0.0001

The cartilage-specific staining including safranin O/Fast green staining, toluidine blue staining and COL II staining were performed to evaluate the ingredient of regenerated tissue. As demonstrated by Fig. 7, the HA+PRP-treated group exhibited stronger safranin O and toluidine blue staining compared to the control groups, suggesting abundant GAG content distributed in the ECM of repaired tissue. Moreover, the HA+PRP-treated group demonstrated best cartilage continuity. The consistent results were observed in the COL II immunohistochemical staining (Fig. 8), demonstrating that the HA+PRP-treated group exhibited stronger COL II expression than the control groups no matter in the FT cartilage defects or osteochondral defects. Compared to the results of COL I immunohistochemical staining (Fig. 9A–D), the type II collagen of HA+PRP-treated group predominated the ECM, indicating that the repaired cartilage was composed of hyaline-like cartilage predominately instead of fibrocartilage, without formation of hypertrophic cartilage as shown by COL X immunohistochemical staining (Fig. 9E–H). For osteochondral defects, the subchondral bone of HA+PRP-treated group was abundant in COL I and COL X, exhibiting good subchondral bone remodeling. Moreover, as shown in Fig. 10A, the HA+PRP-treated group exhibited significantly higher ICRS visual histological score than the HA treated and Blank group, indicating that the HA+PRP-treated group represented the best cartilage repair from histological view compared to other groups.

### Biomechanical test

The quantitative nanoindentation test was performed to assess the biomechanical properties of repaired tissue in terms of reduced modulus and hardness. As demonstrated by Fig. 10B and C, for 8.5 mm FT cartilage defect group, the reduced modulus and hardness (5.21 ± 1.32 GPa; 0.23 ± 0.14 GPa) of HA+PRP treated group were significantly higher than that of Blank group (0.34 ± 0.01 GPa; 0.02 ± 0.01 GPa). For 6.5 mm FT cartilage defect group, the reduced modulus and hardness (7.78 ± 2.15 GPa; 0.23 ± 0.12 GPa) of HA+PRP=treated group were significantly higher than that of Blank group (2.98 ± 0.29 GPa; 0.13 ± 0.01 GPa). For the osteochondral defect group with 8.5 mm in diameter and 5 mm in depth, the reduced modulus and hardness (3.81 ± 1.30 GPa; 0.20 ± 0.04 GPa) of HA+PRP-treated group were significantly higher than that of Blank group (0.19 ± 0.04 GPa; 0.01 ± 0.01 GPa). For the osteochondral defect group with 6.5 mm in diameter and 5 mm in depth, the reduced modulus and hardness (4.94 ± 0.56 GPa; 0.24 ± 0.03 GPa) of HA+PRP-treated group were significantly higher than that of Blank group (2.38 ± 0.56 GPa; 0.07 ± 0.02 GPa). The reduced modulus and hardness of HA+PRP-treated group showed no significant differences with the normal cartilage.

## Discussion

Our study has first investigated the effectiveness of autologous PRP combined with HA hydrogel on the repair of FT cartilage and osteochondral defects with different critical sizes in the medial femoral condyle of a porcine model. The HA+PRP-treated group demonstrated smooth cartilage surface, well integration to adjacent cartilage, improved mechanical properties and columnar chondrocytes distribution with transparent hyaline-like cartilage formation and improved content of GAG and COL II and without formation of hypertrophic cartilage as well as intact subchondral bone trabecular reconstruction. Moreover, the FT cartilage defects with 8.5 mm in diameter and osteochondral defects with 6.5 mm in diameter and 5 mm in depth exhibited superior regeneration treated by the combination of autologous PRP and HA hydrogel. However, the HA hydrogel alone or spontaneous healing obtained inferior cartilage repair in this experiment. The key factors leading to superior cartilage repair may be related to the combined utilization of autologous PRP and HA hydrogel.

The HA hydrogel provided architectural matrix for cell adhesion, migration, proliferation and differentiation. This facilitated the migration of mesenchymal stem cells [13] and cartilage stem cells [14] as well as differentiation toward chondrogenesis or osteogenesis to promote cartilage repair and subchondral bone reconstruction. This may be owing to the following properties of HA hydrogel including (i) highly hydrated microenvironment allowing for improved exchange of substances; (ii) simulating structure of extracellular cellular matrix of native cartilage to facilitate cell–matrix contacts; (iii) well integration to adjacent tissue and (iv) recruitment of mesenchymal stem cells owing to its CD44 targeting moiety [11, 15]. Unlike the porous scaffolds, the HA hydrogel exhibited highly cross-linked structure as demonstrated by Fig. 1B, thus generating a rounded cell shape rather than the excessive spreading shape, inducing chondrogenic differentiation to the maximum extent [16, 17]. Meanwhile, the HA molecule was capable of promoting the chondrogenesis of mesenchymal stem cells [18, 19]. However, in our study, we observed that the application of HA hydrogel alone obtained inferior cartilage repair with rough cartilage surface, inferior integration with adjacent cartilage, lower content of GAG and COL II. The lack of additional growth factors, which would facilitate the progression of chondrogenesis, may account for these unfavorable repair outcomes.

Autologous PRP played an essential role in providing various concentrated endogenous growth factors, which were responsible for potentially facilitating chondrogenesis and cartilage repair [20, 21]. This may lead to the superior repair outcomes when the defects were treated with HA hydrogel combined with autologous PRP. Moreover, the following advantages may accelerate the application of PRP in clinic including the simple preparation of autologous PRP and easy operation using the commercial extraction suit as well as its safety [22] without considering immunoreaction unlike the exogenous growth factors. Some studies have reported that the catabolic and inflammatory cytokines (i.e. tumor necrosis factor and interleukin) that were already existing in prepared PRP or caused by increased concentration of leukocyte in PRP [23] may destroy the ECM of cartilage. While, this phenomenon was absent in the HA+PRP-treated group, which may be associated with the anti-inflammatory effect of HA [24].

The present clinical options for the treatment of chondral defects include the microfracture, osteochondral autograft/allograft transplantation or autologous chondrocyte implantation (ACI) [25]. Each technique has its own appropriate clinical applicability exhibiting that the microfracture technique was suitable for the cartilage lesions less than 2.5 cm^2^, the osteochondral autograft transplantation was used for the lesions from 1 to 4 cm^2^, the ACI technique can be used to treat lesions 2–10 cm^2^ and the osteochondral allograft transplantation may be used to treat larger lesions [26, 27]. Thus, ensuring the precise clinical applicability of cartilage repair technique played an important role in the treatment of various cartilage lesions. However, most of the current animal researches on cartilage repair mainly focused on the single defect size, single defect type (i.e. FT cartilage defect or osteochondral defect) or nonweight bearing area defect [28, 29]. Our study has investigated the effectiveness of autologous PRP combined with HA hydrogel on the repair of FT cartilage and osteochondral defects with different critical sizes in the medial femoral condyle of a porcine model. For FT cartilage defects, the HA+PRP-treated group exhibited superior macroscopic appearance, biomechanical properties and histological evaluation in both 6.5 and 8.5 mm diameter defects. For osteochondral defects, the HA+PRP-treated group exhibited synchronous regeneration of superficial cartilage and subchondral bone in 6.5 mm diameter, 5 mm depth osteochondral defects (Fig. 8D), while, the 8.5 mm diameter, 5 mm depth osteochondral defects exhibited inferior regeneration. As demonstrated by Fig. 8C, the hyaline-like cartilage abundant in Col II appeared in the subchondral bone area, then replaced by bone tissue rich in Col I (Fig. 9C) and Col X (Fig. 9G). Yang *et al.* [30] used the genetic recombination animal model to testify that the hypertrophic chondrocytes can become osteoblasts and osteocytes during endochondral bone formation. Based on this study, we hypothesized that the subchondral bone reconstruction underwent the process of hyaline-like cartilage formation into hypertrophic cartilage then osteogenesis. 

The effectiveness of the combined utility of injectable HA hydrogel and autologous PRP on cartilage repair has been investigated in this study. However, further studies should be performed to facilitate the translation of animal results into clinic, including (i) the comparison between weight bearing and nonweight bearing cartilage repair outcomes, (ii) the repair outcomes combined with early rehabilitation exercises after surgery and (iii) the comparison of different ages and gender of animal model. Until now, many studies on the cartilage repair were based on the short-term researches of small animals [31, 32], thus lacking the long-term assessment of therapeutic effect, which played an important role in the evaluation of cartilage regeneration. Furthermore, the kissing lesions may occur in the corresponding area of tibial plateau due to the absence of early effective immobilization on large animals. However, the kissing lesions can be prevented on humans under the circumstance of early immobilization and appropriate rehabilitation. Thus, we have the reason to believe that the clinical results may be superior to the animal results.

## Conclusions

Our study has first verified the efficacy of autologous PRP combined with injectable HA hydrogel on the regeneration of FT cartilage defects and osteochondral defects with different sizes in the weight bearing tibiofemoral joint of a porcine model. The FT cartilage defect with 8.5 mm in diameter and osteochondral defect with 6.5 mm in diameter and 5 mm in depth exhibited superior regeneration treated by the combination of HA hydrogel and autologous PRP, indicating its potential in future clinical application.
